# Preparing for the end-of-life: public attitudes towards advance directives and assisted suicide in Switzerland

**DOI:** 10.1186/s12904-025-01758-7

**Published:** 2025-04-26

**Authors:** Solenne Blanc, Clément Meier, Claudia Gamondi, Jürgen Maurer, Ralf J. Jox

**Affiliations:** 1 Palliative and Supportive Care Service, Chair in Geriatric Palliative Care, and Institute of Humanities in Medicine, Lausanne University Hospital and University of Lausanne, Avenue Pierre-Decker 5, CH- 1011 Lausanne, Switzerland; 2 Faculty of Business and Economics (HEC), University of Lausanne, and Swiss Centre of Expertise in the Social Sciences (FORS), Swiss National Centre of Competence in Research LIVES – Overcoming vulnerability: Life course perspectives, Lausanne, Switzerland; 3 Palliative and Supportive Care Service, Chair in Geriatric Palliative Care, Lausanne University Hospital and University of Lausanne, Lausanne, Switzerland; 4 Faculty of Business and Economics (HEC), University of Lausanne, Lausanne, Switzerland

**Keywords:** Assisted dying, Palliative care, Advance care planning, Patient preferences, Advance directives

## Abstract

**Background:**

Despite public support for assisted suicide (AS) and advance directives (AD), no studies have established whether individuals express interest in both procedures. This study investigates the relationship between AD completion and attitudes toward assisted suicide, examining whether Swiss older adults inclined toward AD also demonstrate positive attitudes toward AS.

**Method:**

Data from 1,523 participants aged 58 and older were collected through the Swiss component of the representative Survey on Health, Ageing, and Retirement in Europe (SHARE) for 2019/2020. Participants were asked if they had completed AD. Attitudes toward AS were assessed using three key questions: support for AS, consideration of it, and membership in a right-to-die organization. Probit regression models analyzed the associations, considering various social, health, and regional characteristics.

**Results:**

Overall, 42% of the sample had completed AD. Additionally, 81% supported legal access to AS, 63% considered asking for it, and 9% were members of a right-to-die association. Among members of a right-to-die a, 89% had completed an AD. Respondents who had completed AD were more likely to support AS (*p* < 0.001), consider it (*p* < 0.001), and be members of a right-to-die organization (*p* < 0.001).

**Conclusions:**

The study reveals an association between completing AD and supporting attitudes toward AS among older adults in Switzerland, highlighting how the desire to control end-of-life experiences can drive interest in both procedures. Future developments in end-of-life care planning should consider incorporating discussions and documentation of AD and AS together.

**Supplementary Information:**

The online version contains supplementary material available at 10.1186/s12904-025-01758-7.

## Introduction

As they face multiple hospitalizations, older adults are often requested to proactively address future healthcare challenges by expressing their preferences for care settings, medical interventions, or treatment plans [1, 2]. Such challenges are commonly discussed in end-of-life planning, via participation in Advance Care Planning (ACP), and can be documented in Advance Directives (AD) [3, 4]. ACP is a process that involves discussing and documenting an individual’s healthcare preferences to ensure that future treatment aligns with their preferences and goals of care if they become unable to decide [5–7]. Decisions made during ACP can ultimately be documented in AD forms [8]. AD enables individuals to specify their treatment preferences in writing and designate a healthcare proxy to make decisions on their behalf if they are unable to do so themselves [9]. There is a growing body of literature that recognizes the importance of AD in safeguarding patient autonomy, ensuring treatments align with personal wishes, and guiding families and healthcare providers in difficult medical decisions [10].

AD forms typically include information on healthcare preferences, end-of-life care, designation of a healthcare proxy, and personal values. However, worldwide, various forms of AD exist, and their content can vary depending on countries’ regulations. In addition, previous studies have demonstrated that individuals tend to engage in the writing of AD rather variably: across Europe, completion rates range from 0 to 77% [11]. In Switzerland, while federal regulations for AD were established in 2013, support for their writing relies on federal promotion and recommendations through the publication of guidelines for ACP [12]. In this context, various methods for documenting AD exist, making it challenging to assess uniform national completion rates. Two studies have, however, reported rates based on national samples. Among individuals aged 55 and older, the completion rate of advance directives (across all models) was estimated at approximately 20% in 2015 [13], whereas a 2019 study reported a completion rate of 42% among adults aged 58 and older [14]. Although a significant increase in interest could not be demonstrated, these studies concluded that factors such as education, older age, and higher health literacy increase the likelihood of engaging in end-of-life care planning and completing an AD.

When going through the process of writing AD, individuals are invited to express and discuss their preferences regarding end-of-life care. Among available options, older adults may mention an interest in assisted dying – a term that designates practices such as euthanasia or physician-assisted suicide [15] – and seek clarification on related practices, as evidence points to growing public support for these practices across various jurisdictions in Western Europe [16]. In Switzerland, while euthanasia is prohibited, assisted suicide (AS) – defined as the self-administration of a lethal drug [17] — is permitted under specific conditions and supported by right-to-die organizations. These organizations assist by organizing medical documents, obtaining the prescription for the lethal drug, ensuring compliance with legal criteria, and accompanying the individual during the self-administration of the lethal drug [18]. Over the past decades, membership in right-to-die organizations and the number of requests for assistance have increased in Switzerland, and worldwide [19].

As evidenced by rates of AD completion and memberships in right-to-die organizations, Swiss older adults seem to express a growing interest in both AD and AS. Whilst research has examined individuals’ support for both procedures in parallel, to our best knowledge, there have been no studies establishing whether these individuals might demonstrate positive support towards both procedures simultaneously. Hence, this study investigates the association between the completion of AD and attitudes toward AS among older adults in Switzerland, evaluating if older adults might be inclined to engage in AD and, at the same time, support AS. More specifically, it aims to determine if an association exists between the two, investigating the extent to which completing AD is associated with positive attitudes toward AS.

## Methods

### Study design and participants

The data used originated from 1,523 adults aged 58 years and older, living in Switzerland, who participated in a paper-and-pencil questionnaire as part of Wave 8 of the Swiss component of the Survey on Health, Ageing, and Retirement in Europe (SHARE). SHARE is a comprehensive longitudinal study spanning 27 European countries and Israel [20, 21]. The SHARE target population consists of all persons aged 50 years and over at the time of sampling who have their regular domicile in Switzerland. Individuals are excluded if they are incarcerated, hospitalized, out of the country during the entire survey period, unable to speak the country’s language(s), or have moved to an unknown address. The sample is drawn using probabilistic methods, with stratification based on geographic regions and sex to ensure national representativeness [22]. Every two years, it gathers detailed data through face-to-face interviews to provide insights into the health, socioeconomic status, and social or family networks of individuals aged 50 years and older, including their partners. Data collection was conducted between October 2019 and March 2020. During Wave 8, a total of 2,005 Swiss respondents and their partners took part in in-person interviews, with 1,891 (94.3%) also completing the additional national paper-and-pencil questionnaire. For the purposes of this study, the analysis focused on respondents aged 58 years and older. Although SHARE is designed to be representative of individuals aged 50 and older, the Swiss sample has not been refreshed since 2011. As a result, by Wave 8 (2019/2020), it was only representative of those aged 58 +. While some individuals under 58 remained in the dataset, they were exclusively the partners of previously sampled respondents and were not systematically recruited. To ensure consistency and preserve the representativeness of the sample, we excluded these younger partners from the analysis. Moreover, respondents who did not answer all questions relevant to the analysis were excluded. Exclusion was determined by incomplete or partial questionnaire responses. The leading causes of non-completion could include fatigue associated with aging or illness. Consequently, the effective sample size for the study was narrowed down to 1,523 participants.

### Outcome variable

#### Attitudes toward AS

In the paper-and-pencil questionnaire, participants were asked in a specific part about their attitude about legal access to AS as per the current laws in Switzerland (see Additional file 1). They were asked whether they support the legality of AS as it is currently the case in Switzerland, with response options including “Yes” or “No”. Furthermore, participants were questioned if they could imagine circumstances under which they would consider asking for AS themselves. Response options included “Yes” and “No”. Finally, participants were asked if they were a member of any right-to-die organization with possible response options including “Yes” and “No”.

### Exposure

#### Completion of AD

Another question from the paper-and-pencil questionnaire asked participants about their engagement with AD (see Additional file 1). Participants were asked whether they had completed a written form of AD, with possible response options, including “Yes” and “No.”

### Covariates

Our statistical models incorporate various demographic variables, including sex (male, female), age groups (58–64 years, 65–74 years, 75 + years) [23], educational attainment (low = International Standard Classification of Education (ISCED) levels 0–1–2, middle = ISCED levels 3–4, high = ISCED levels 5–6), partnership status (has a partner, has no partner), subjective financial difficulties (assessed by ability to make ends meet: easily, fairly easily, with difficulty, linguistic regions within Switzerland (German, French, or Italian), living area (urban, rural), and self-rated health (categorized as poor/fair health, good health, very good/excellent health).

### Statistical analysis

The study population’s characteristics were described through numerical counts and proportions. Pie charts were used to present the distribution of AD completion and attitudes toward AS. Then, bar charts were utilized to analyze the correlation between the percentages of respondents with AD and the types of attitudes toward AS. Probit regressions were conducted to assess the partial association between AD completion and attitudes toward AS. Each regression model controlled for several variables, including sex, age, education levels, partnership status, subjective financial difficulties, Switzerland’s linguistic regions, living areas, and self-rated health. All analyses were conducted using STATA/SE 18.0 software (STATA Corporation, College Station, TX), and results were presented as average marginal effects (AME) with corresponding standard errors (SE) clustered at the household level to address potential unobserved dependencies between the target respondents and their partners.

## Results

The demographic characteristics of the study participants are presented in Table 1. Among the sample 52.1% self-identified as female and 47.9% as male. Concerning age groups, the study population was divided into three categories, with 26.9% of individuals aged 58–64 years, 40.9% aged 65–74 years, and 32.2% aged 75 years or older. When considering education levels, most participants reported having a middle level of education (62.8%), followed by those with a high level of education (20.9%), and those with a low level of education (16.3%). Partnership status indicated that 75.3% of respondents had a partner, while 24.7% did not. Financially, 55.9% of participants reported being able to make ends meet easily, 30.9% could do so fairly easily, and 13.2% experienced difficulty in making ends meet. The majority spoke German (72.4%), followed by French (24.2%), and Italian (3.4%). The sample was close to evenly split between urban (45.9%) and rural (54.1%) areas. Self-rated health varied, with 17.4% considering their health to be poor or fair, 43.0% rating it as good, and 40.6% as very good or excellent.

**Table 1:** Characteristics of the study population, adults aged 58 +, SHARE Switzerland, 2019/2020, *n* = 1,523

	*n*	%
**Sex**		
Male	729	47.9
Female	794	52.1
**Age groups**		
58–64 years	409	26.9
65–74 years	623	40.9
75 + years	491	32.2
**Education**		
Low	248	16.3
Middle	956	62.8
High	319	20.9
**Partnership status**		
Has a partner	1,147	75.3
No partner	376	24.7
**Make ends meet**		
Easily	851	55.9
Fairly easily	471	30.9
With difficulty	201	13.2
**Language**		
German	1,102	72.4
French	369	24.2
Italian	52	3.4
**Living area**		
Urban	699	45.9
Rural	824	54.1
**Self-rated health**		
Poor/fair health	265	17.4
Good health	639	43
Very good/excellent health	619	40.6
**Completed AD**		
No	877	57.6
Yes	646	42.4
**Supports assisted suicide**		
No	287	18.8
Yes	1,236	81.2
**Consider asking for assisted suicide**		
No	562	36.9
Yes	961	63.1
**Member of right-to-die organization**		
No	1,384	90.9
Yes	139	9.1

Number of observations for the whole sample

**Fig. 1:**
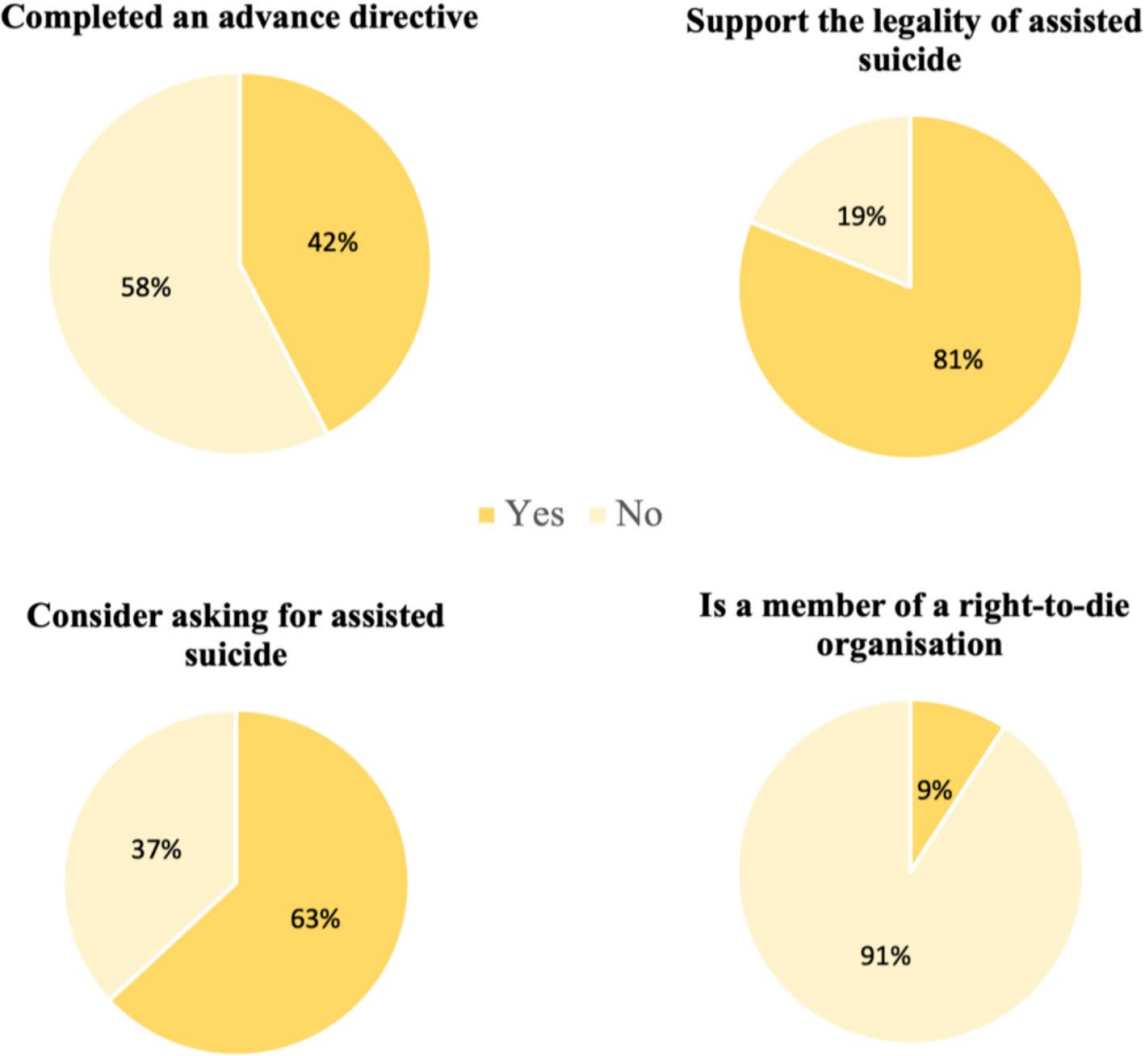
Distribution of advance directives completion and attitudes toward assisted suicide, adults aged 58 +, SHARE Switzerland, 2019/202, *n* = 1,523

Figure 1 illustrates the distribution of AD completion and attitudes toward AS. Regarding completion of AD, 42% of participants had done so, while 58% had not. The majority of respondents (81%) stated they supported the legality of AS as it is currently the case in Switzerland, a significant portion (63%) reported considering asking for AS themselves and 9% reported being members of right-to-die organizations.

**Fig. 2:**
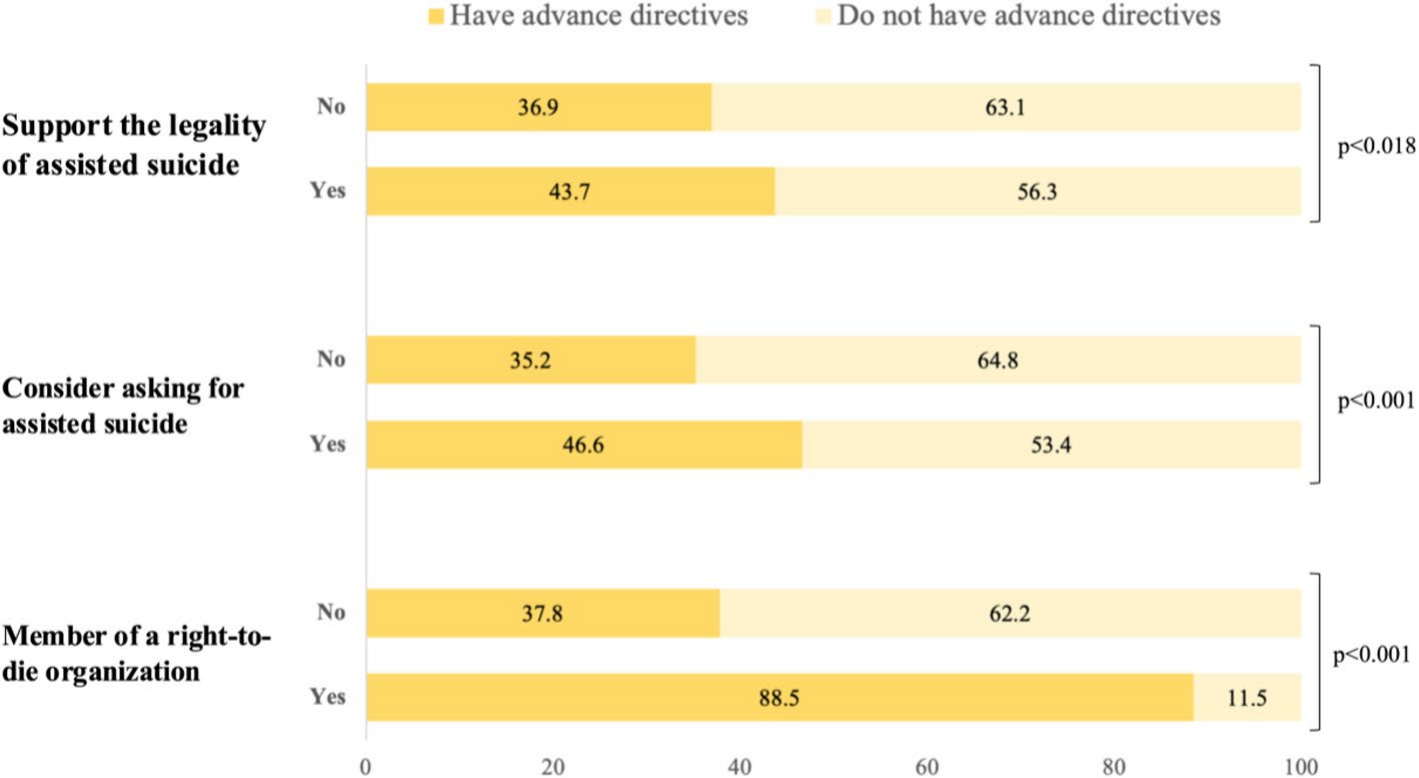
Percentage of respondents with advance directives by types of attitudes toward assisted suicide, adults aged 58 +, SHARE Switzerland, 2019/2020, *n* = 1,523

Figure 2 illustrates the percentage of respondents with AD compared to those without, grouped according to three types of attitudes toward AS. A statistical test (e.g., chi-square test) was conducted to assess the significance of differences in AD completion across these groups. This figure provides three key results: (1) an overall comparison of AD completion across the three attitude types about the support for the respective attitudes, sorted by positive (“Yes”) or negative (“No”) responses, (2) the distribution of AD completion within the subgroup supporting the related attitude (i.e. those who answered “Yes”), (3) the distribution of AD completion within the subgroup not supporting the related attitude (i.e. those who answered “No”).

Across all three types of attitudes and comparing the proportions of AD completion between subgroups (supportive vs non-supportive), individuals who express support for AS tend to have higher rates of AD completion compared to those expressing negative support; for instance, respondents’ AD completion rates in memberships indicates 88% of completion among members compared to 37.8% among non-members. A similar trend is observed across the two other attitudes. Overall proportions in legality of AS attitude depict higher rates of respondents not possessing AD than those having them: within the supporting subgroup, the distribution illustrates a smaller proportion of respondents with AD (43.7%) than those without them (56.3%). Likewise, among respondents who do not support the legality of AS, 36.9% have AD, while 63.1% do not. The observed difference within subgroups is statistically significant (*p* < 0.018). Similarly, higher rates of AD non-completion are depicted in subgroups related to consideration for AS. The subgroup showing positive consideration is divided into 53.4% individuals not possessing AD and 46.6% who do. Respondents who would not consider asking for AS also report a higher proportion of AD non-completion (64.8% of respondents did not complete AD, and 35.2% indicated having completed them). This difference is also statistically significant (*p* < 0.001). Comparing subgroups demonstrates that respondents who would consider asking for AS seem more likely to have AD than those who show negative support. Memberships show a different proportion distribution: among members, 88.5% have written their AD and 11.5% have not, meaning the subgroup representing positive support shows a significant majority of completion. In contrast, those who are not members of such an organization show lower rates of AD completion (37.8%), with a majority not having AD (62.2%). This difference is statistically significant (*p* < 0.001). When comparing the distribution of the subgroups, respondents supporting positively the legality of AS are more likely to have AD than those who do not support it. Individuals with AD consistently demonstrate a higher propensity to support the legality of AS, consider asking for AS, and join a right-to-die organization.

Table 2 displays the partial associations between the completion of AD, attitudes toward AS, and various covariates. The data show that being 75 years or older compared to being between 58 and 64 years is associated with a decrease in the likelihood of supporting AS (AME: − 0.08, *p* < 0.05), and considering asking for AS (AME: − 0.16, *p* < 0.001). Additionally, individuals without a partner compared to those with a partner are less likely to support AS (AME: − 0.05, *p* < 0.05) and consider asking for AS (AME: − 0.08, *p* < 0.05). Speaking Italian compared to German is linked to a reduced likelihood of supporting AS (AME: − 0.20, *p* < 0.05). Having tertiary or secondary levels of education compared to a primary one increased the likelihood of supporting AS (AME: 0.09, *p* < 0.05). Individuals who rate their health as very good or excellent compared to bad health are more likely to support AS (AME: 0.07, *p* < 0.05). Finally, individuals who have completed AD are significantly more likely to support AS (AME: 0.07, *p* < 0.01), consider asking for AS (AME: 0.15, *p* < 0.001), and being a member of a right-to-die organization (AME: 0.20, *p* < 0.001).

**Table 2:** Associations between attitudes toward assisted suicide, advance directives and covariates, adults aged 58 +, 2019/2020, *n* = 1,523

	**Support assisted suicide**	**Consider asking for assisted suicide**	**Member of right-to-die organization**
**Sex** (male)			
female	0.00	− 0.04	0.02
	(0.02)	(0.02)	(0.01)
**Age group** (58–64 years)			
65–74 years	0.01	− 0.04	0.03
	(0.03)	(0.03)	(0.02)
75 + years	− 0.08^**^	− 0.16^***^	0.00
	(0.03)	(0.04)	(0.02)
**Partnership status** (has a partner)			
no partner	− 0.05^*^	− 0.08^*^	− 0.00
	(0.03)	(0.03)	(0.02)
**Language** (German)			
French	0.02	0.01	0.10^***^
	(0.02)	(0.03)	(0.03)
Italian	− 0.20^**^	− 0.13	0.07
	(0.08)	(0.08)	(0.08)
**Education** (primary)			
secondary	0.06^*^	0.01	0.00
	(0.03)	(0.03)	(0.02)
tertiary	0.09^*^	0.04	0.04
	(0.04)	(0.04)	(0.03)
**Make ends meet** (easily)			
fairly easily	0.01	− 0.02	− 0.02
	(0.02)	(0.03)	(0.02)
with difficulty	− 0.03	− 0.01	− 0.02
	(0.03)	(0.04)	(0.02)
**Living area** (urban)			
Rural	0.01	− 0.02	0.01
	(0.02)	(0.03)	(0.02)
**Self-rated health** (bad health)			
good health	0.02	− 0.02	− 0.01
	(0.03)	(0.03)	(0.02)
very good/excellent health	0.07^*^	0.01	− 0.01
	(0.03)	(0.04)	(0.02)
**Completed AD (No)**			
Yes	0.07^**^	0.15^***^	0.20^***^
	(0.02)	(0.03)	(0.02)
Observations	1523	1523	1523

The table shows average marginal effects and standard errors in parentheses. Statistical significance: * *p* < 0.05, **p < 0.01, *** *p* < 0.001. The three columns present probit regressions models regressing each attitude toward assisted suicide on advance directives completion and the covariates. The covariates include sex, age, education levels, partnership status, subjective financial situation, linguistic region, living area and self-rated health

## Discussion

The results of the present study indicate that two-fifths of the sample have completed AD, over 80% support the legality of AS, 62% might consider it in the future and a tenth of respondents are members of a right-to-die association. In addition, results show that individuals who completed AD are also more likely to express favorable attitudes towards AS, measured as support for the legality of AS in Switzerland, consideration in asking for AS themselves, and memberships of right-to-die organizations. The most significant finding is the positive and statistically significant association between these two procedures. A closer examination of the factors that may potentially explain this relationship is required, several of which will be elaborated upon in the subsequent paragraphs.

### The influence of socio-demographic characteristics on attitudes towards AS

Our study illustrates that demographic factors such as age, partnership status, language, education level, and self-rated health factors are associated positively with attitudes toward AS. Consideration for the impact of these factors on interest for AS is necessary. Interestingly, consideration for AS yields significantly decreasing chances of showing positive attitudes among adults over 75. One study concluded that a generational gap exists in attitudes toward end-of-life decision-making, suggesting that the tendency to assert control over such decisions may have emerged in the mid-1990s [24]. In addition, numerous studies have shown that with increasing age comes prioritization of intrinsic values [25], finding stronger meaning in factors related to family, partnership, and spirituality over health, pleasure, and work [26]: older adults may thus reassess their inclination for this option as they age. This hypothesis may strongly resonate in the Swiss context, where memberships can be acquired upon Swiss residency and legal age at little cost, a practice that does not necessarily lead to requesting AS – which would also explain why both young and older individuals acquire memberships without showing significant differences in attitudes – then reconsider this inclination by prioritizing intrinsic values.

While studies generally suggest that having a partner decreases the likelihood of considering AS as an end-of-life option [27], our results indicate the opposite effect. A possible explanation for this discrepancy might lie in the perception of being a burden, which has been identified as one among multiple factors influencing AS requests in partnered individuals [28]. Therefore, not having a partner might serve as a protective factor against this motivation, leading to lower support for AS. However, our results may also be influenced by the characteristics of our sample, which is predominantly composed of individuals with a partner.

The third variable that shows a significant and positive association is language, indicating that Italian-speaking participants are less likely to support AS while German-speaking individuals are more likely to be members of organizations. This finding can be explained by cultural background differences between Swiss regions. However, it is also probably affected by the disparity in subgroup sizes within our sample, which comprises 1,100 German-speaking individuals, whereas the Italian-speaking group consists of only 50 participants.

Finally, previous findings indicate that healthy, educated older adults with higher health literacy are more likely to engage in end-of-life care planning [14], a trend that aligns with our findings.

### Personal autonomy and end-of-life care choices

The significant association between both procedures evidences a shared interest among individuals and contributes to the understanding of a potential increase in social inclination toward end-of-life decision-making practices. A possible explanation may lie in the desire for self-determination and autonomy when making end-of-life decisions. Research has shown that wanting to maintain control over care and a stronger advocacy for personal autonomy are key drivers for individuals to engage in ACP [29] and consider AS [30]. Therefore, older individuals who prioritize autonomy in end-of-life care decisions could be more likely to pursue both procedures. Our findings highlight that both completion of AD and support of AS may reflect an intention to maintain control over end-of-life decision-making – from specifying preferences for healthcare treatments to deciding on the time and place of death. Thus, for individuals wishing to maintain control over their end-of-life decisions, AD and AS can be seen as complementary options.

### The interplay between AD and AS decisions

The positive association between AD and AS could be interpreted in different ways. On one hand, it may suggest that individuals who initially completed AD were more likely to consider assisted suicide as an option later on. However, it is important to acknowledge that the association might also go in the opposite direction – joining a right-to-die organization may motivate individuals to complete AD. Individuals might then first become members of these organizations, then complete their AD as a precaution, in case they lose mental capacity. This phenomenon is comparable to the “fear of losing the window”, the “five minutes to midnight” or “5 min to 12” effect that has been identified in other research studying the interplay between assisted dying challenges and AD [31]. Therefore, individuals may prioritize engagement in assisted dying procedures, while viewing AD as a form of “double security”. According to this interpretation, Swiss older adults might prioritize supporting AS over completing AD. In addition, until 2021, some right-to-die organizations in Switzerland provided their own AD templates and offered them to all of their new members [19], which could explain the high AD completion rates among observed memberships.

### Practical implications: a complementary procedure?

The significant number of older adults with AD and supporting AS observed in this study suggest that individuals who completed AD may have considered AS as an option for their end-of-life. This finding provides important insights for end-of-life care planning, with considerations that extend beyond the Swiss context. Beyond variations across jurisdictions in the implementation of AS and AD procedures, research related to the umbrella term of assisted dying provide valuable insights into practical implications of this finding. For example, White et al. (2024) have demonstrated that many individuals expect to engage in discussions regarding assisted dying potentiality with their physicians [32]. These considerations underscore the importance for clinicians to engage in informed discussions when individuals present with such inquiries, ensuring that patients are well-informed about end-of-life available options. While ethical and clinical concerns may arise, research indicates that the preference for assisted dying remains stable over time [33] and that discussing these matters clarifies potential misconceptions regarding the distinctions between AD and assisted dying [34, 35]. Hence, ACP discussions constitute an ideal opportunity to address related concerns about assisted dying, ensuring that patients’ preferences are fully considered. Several countries have already made advancements in this area. For instance, Belgium and the Netherlands allow individuals to include their preferences for physician-assisted dying in their AD [26, 27], and studies have identified that, among individuals who completed AD, a significant proportion possess physician-assisted dying AD [36, 37].

In Switzerland, the individual interest in AS has not been formally integrated into AD; they remain two independent procedures with distinct objectives, each driven by personal motivations and engagement. Although AS, in contrast to euthanasia, is not amenable to an anticipatory decision by AD, the observed shared interest in both procedures – as seen in this study through the positive association, which could be explained by the desire to enhance self-determination – warrants the reflection about a converging approach. This interpretation, however, calls for further ethical considerations and discussions to evaluate the significance of ACP practices in the context of an integrated approach and the adaptation of AD documents in Switzerland.

### Strengths and Limitations

This study has several strengths but also acknowledges its limitations. First, the questions about AD completion and attitudes toward AS may not fully capture the complex and nuanced nature of end-of-life planning and preferences. Factors such as personal or family experiences with terminal illness, cultural values, or religious beliefs, which could significantly influence these decisions, were not directly measured, but have been explored in previous studies [24]. Additionally, acquiring membership in a right-to-die organization in Switzerland may not always reflect active end-of-life decision-making but could instead serve as a precautionary measure or symbolic support for the organization’s values. While our sample is based on a nationally representative dataset, the findings on the prevalence of advance directives completion and attitudes toward assisted suicide should be interpreted with caution, as they may not fully reflect the current national prevalence. Nevertheless, the study’s strength lies in its ability to examine the associations between these variables rather than estimating precise prevalence rates. There is also a risk of underrepresentation of the vulnerable members of the older population due to attrition, and missing responses may have introduced bias. However, the study mitigates these issues through a high response rate and demographic consistency between included and excluded participants, which strengthens the validity of the findings. The study’s high response rate and probabilistic sampling design enhance the reliability of the data. While the cross-sectional nature of the study limits the ability to establish causal relationships between AD and attitudes toward assisted dying, it still provides valuable insights into associations that can serve as a foundation for future research. Exploring the associations between AD completion and attitudes toward assisted suicide among older adults in Switzerland provides additional understanding of end-of-life decision-making. Further qualitative research is needed to assess the significance attributed to these associations and to develop a more comprehensive understanding of this phenomenon. These strengths, combined with the study’s relevance to the discourse on personal autonomy and end-of-life choices, ensure that the findings are valuable for both healthcare professionals and policymakers involved in advance care planning and assisted dying practices.

## Conclusion

By identifying a positive and significant association between AD completion and support for AS, this study contributes to our understanding of older adults’ support for end-of-life healthcare decisions. It participates in future developments in end-of-life care planning, emphasizing the potential need to integrate discussions and documentation of AD in Switzerland, following other countries’ complementary approaches. ACP discussions represent a good alternative, and healthcare professionals should pay particular attention to individuals who might consider AS an end-of-life decision. Hence, practitioners should be prepared to engage in discussions providing information about the rights, requirements, and conditions under which AS is permitted. Integrating discussions and documentation of both AD and AS in ACP could enhance patient autonomy and ensure that end-of-life care aligns with individual preferences and values. This approach may lead to more informed and compassionate care, ultimately improving the quality of life for older adults.

## Supplementary Information

Additional file 1: Swiss component of the Survey on Health, Ageing, and Retirement in Europe (SHARE), questions from the paper-pencil questionnaire. Appendix 1 and Appendix 2

## Data Availability

This paper uses data from SHARE-ERIC (2024). Survey of Health, Ageing and Retirement in Europe (SHARE) Wave 8. Release version: 9.0.0. SHARE-ERIC. Data set. DOI: 10.6103/SHARE.w8.900. Study data already de-identified are available to the scientific community upon submitting a data requestion application to the SHARE study.
