# Supplementary material for: Preparing for the end-of-life: public attitudes towards advance directives and assisted suicide in Switzerland
Source: BMC Palliat Care. 2025 Apr 26;24:118. doi: 10.1186/s12904-025-01758-7 (PMC12034111; doi:10.1186/s12904-025-01758-7)
Supplement: Supplementary file 1 — Additional file 1: Swiss component of the Survey on Health, Ageing, and Retirement in Europe (SHARE), questions from the paper-pencil questionnaire. Appendix 1 and Appendix 2 [file 12904_2025_1758_MOESM1_ESM.docx]

**Swiss component of the Survey on Health, Ageing, and Retirement in Europe (SHARE), questions from the paper-pencil questionnaire**

| **Appendix 1: Attitudes toward assisted suicide** |
| --- |
| **Question 1:** Do you support the legality of assisted suicide as it is currently the case in Switzerland?  Answer: “Yes” or “No”  **Question 2:** Can you imagine circumstances under which you would consider asking for assisted suicide yourself?  Answer: “Yes” or “No”  **Question 3:** There are associations in Switzerland, such as “Exit” or “Dignitas”, that offer assistance in suicide. Are you a member of such an association?  Answer: “Yes” or “No” |

| **Appendix 2: Completion of advance directives** |
| --- |
| **Question 1:** Advance directives are a written statement in which an individual can describe his/her preferences for medical treatments and care in case he/she is no longer able to decide by him-/herself. Individuals can also designate someone who can make medical decisions for them if necessary. This written statement is binding for medical providers and relatives.  Have you completed a written statement about your wishes and refusals for medical treatments and care (advance directives)?  Answer: “Yes” or “No” |
